# Prevalence, microbiological profile, and outcomes of spontaneous bacterial and fungal peritonitis in Egyptian patients with liver cirrhosis

**DOI:** 10.1186/s12876-025-04546-y

**Published:** 2026-05-29

**Authors:** Zeinab Seif El-Din, Eman Abdelsameea, Samah Mohammad Awad, Doaa Elsayed Arab, Maha Elsabaawy, Mohsen Salama, Ahmed Nashaat Mohamed

**Affiliations:** 1 Hepatology and Gastroenterology Department, National Liver Institute, Menoufia University, Shebin El-Kom, Egypt; 2 Microbiology and Immunology Department, National Liver Institute, Menoufia University, Shebin El-Kom, Egypt; 3 Resident of Hepatology and Gastroenterology, Shebin El-Kom Educational Hospital, Shebin El-Kom, Egypt; 4 Fellow of Hepatogastroenterology and Endemic Diseases, the National Hepatology And Tropical Medicine Research Institute, Cairo, Egypt

**Keywords:** Spontaneous fungal peritonitis ascites, Hepatic cirrhosis, Spontaneous bacterial peritonitis

## Abstract

**Background:**

One of serious side effects of liver cirrhosis is spontaneous bacterial peritonitis (SBP), which is defined by existence of infected ascites without a discernible secondary source of infection. One possible mechanism for development of SBP is microbial translocation.

**Objective:**

To ascertain prevalence, microbiological profile and outcomes of spontaneous bacterial and fungal peritonitis in Egyptian patients with liver cirrhosis.

**Methods:**

Three hundred cirrhotic patients with ascites who were hospitalized for spontaneous peritonitis (SP) participated in cross-sectional research. Laboratory investigations using bacterial and fungal cultures were carried out.

**Results:**

All participants with diagnosis of SP were included. Their mean age was 59.50 years, and 68.7% of them were men. In addition, 13.7% of subjects had spontaneous fungal peritonitis, whereas 47.3% of participants had SBP. Of SBP cases, 50.7% (*n* = 72) had gram negative (-ve) bacteria isolated from them, whereas 49.3% (*n* = 70) had gram positive (+ ve) bacteria. Streptococcus 8.5% (*n* = 12) was the commenest gram-positive isolate, followed by Staphylococcus aureus 33.8%(*n* = 48). On the other hand, Klebsiella 29.6% (*n* = 42) was the commenest gram-negative isolate, followed by Escherichia coli 14.4%(*n* = 19). The outcome of SBP cases with and without multidrug resistance do not differ significantly. On multivariate analysis, femal (*p* = 0.013), low ascitic protein level and high ascitic lactate dehydrogenase (LDH) (*p* = 0.018, *p* = 0.014) were significant independent predictors for SFP.

**Conclusion:**

SBP and SFP frequently provide serious life-threatening dangers in cirrhotic patients with ascites. Alertness for SFP is increased with low ascitic protein level and high ascitic LDH.

## Introduction

A complex disorder, cirrhosis-associated immune dysfunction syndrome(CAIDS) is typified by systemic immune dysfunction that hinders the bloodstream’s ability to rid itself of bacteria, cytokines, and endotoxins. 90% of the reticuloendothelial cells (RE) cells, which are essential for destroying bacteria and include Kupffer and sinusoidal endothelial cells, are found in the liver [1, 2].

About 32%–34% of hospitalized cirrhosis cases and 45% of cases with gastrointestinal bleeding develop bacterial infections. In hospitalized cases, the detected rates significantly surpass the average general infection rate of 5% to 7%. Urinary tract infections (UTIs) account for 20%, spontaneous bacterial peritonitis (SBP) for 25%, and pneumonia for 15% of all infections [3].

In hospitalized cases with cirrhosis and ascites, the incidence of SBP ranges from ten to thirty percent. About 50% of cases are identified upon hospitalization, while the remaining 50% arise during the hospitalization period [4]. SBP as one of the common infection in hospitalized cirrhotic patients has mortality rate in the range of 20–30% [5]. Klebsiella species, Escherichia (E.) Coli, and other Enterobacteriaceae, streptococci, enterococci, and Pseudomonas aeruginosa are the main culprits behind these illnesses [6].

As long as there is no medically curable intra-abdominal source of infection, abdominal fluid polymorphnuclear cell count of at least two hundreds and fifty cells/µL are required for the diagnosis of SBP [7].

Based on abdominal fluid culture results, neutophil cell count, and clinical setting, ascitic fluid infections are divided into five types: Secondary peritonitis, monomicrobial and polymicrobial bacterial ascites, culture-negative SBP, also referred to as culture-negative neutrocytic ascites (CNNA), and classic culture-positive SBP. Intestinal Gram-negative bacteria are the main causes of SBP [8].

Third-generation cephalosporins are advised as empirical antimicrobial therapy in the current spontaneous peritonitis (SP) management recommendations. This regimen, however, does not resolve SP in 7–17% of cirrhotic cases. A major contributing reason to the failure of antimicrobial treatment is fungal superinfections. Fungi are rare in ascitic specimens and are frequently associated with bacterial coinfection. The commenestly isolated fungus in ascite cultures is Candida species. The abnormal condition known as SFP is still not well described [9].

## Patients and methods

Three hundred cirrhotic individuals with SP who were hospitalized were included in this prospective analysis. Participants were chosen from the National Hepatic Institute’s Menoufia University’s Hepatology and Gastroenterology department’s inpatient unit.

### Ethical consideration

Every participant in the research or their family members gave their informed consent. The research was approved by the Menoufia University-affiliated ethical committee of the National Hepatic Institute.

### Inclusion criteria

Patients with decompenated liver cirrhosis with ascites.

### Exclusion criteria

Ascites with causes other than liver cirrhosis; signs of active infections unrelated to ascitic fluid infection; antimicrobial use before hospitalization; the presence of alternative causes of neutrocytic ascites (hemorrhagic ascites, pancreatitis, peritoneal carcinomatosis, appendicitis, tuberculosis); abdominal surgery within three months before the research; and cases with malignancies (apart from cases of secondary peritonitis and hepatocellular carcinoma) were all excluded.

A systematic approach that includes diagnostic abdominal paracentesis, thorough history taking, abdominal ultrasonography, laboratory testing as complete blood count, liver and renal tests, and a thorough clinical assessment was performed on all research participants. Child Pugh classification and the Model of End Stage Liver Disease (MELD) score were used to determine the severity of hepatic disease. Hepatitis B surface antigen (HBsAg) and hepatitis C virus antibody (HCV Ab) tests were performed to identify the cause of chronic hepatic illness. The National liver Institute’s ethical council at Menoufia University approved the research methodology, and each participant gave written, informed consent.

### Diagnostic abdominal paracentesis

Using a wide-bore needle at the case’s bedside, the paracentesis procedure was explained to them and carried out aseptically. The case was supine when the needle was placed in the lower right quadrant. The “Z Tracking” method was used to stop leaks after paracentesis. Each participant had ten milliliters of ascitic fluid taken, which was then examined in accordance with the stated protocol: Biochemical markers such as albumin, glucose, and lactate dehydrogenase (LDH) were quantified. When polymorphonuclear cell counts surpassed two hundreds and fifty cells/mm³ and there was no clinical or radiological indication of secondary peritonitis (such as gastrointestinal perforation), SBP was diagnosed based on white blood cell counts. Using anaerobic blood culture bottles and Bact/Alert aerobic for all specimens, microbiological cultures were carried out to identify harmful organisms. MacConkey agar and blood agar plates were used for bacterial isolation, whereas Sabouraud dextrose agar and CHROMagar™ Candida medium were used for fungus isolation. The automated Vitek^®^ 2 system and disc diffusion technology were used to evaluate the isolates’ susceptibility profiles to antimicrobials and antifungals, while traditional techniques were used to identify MDR bacteria.

According to EASL guidelines, a case with signs of infection who has an abdominal fluid polymorph nuclear cell count greater than teo hundreds and fifty cells/µL is diagnosed with MDR SBP. This is particularly important if the infection does not respond to first-line empirical antimicrobials, which is a crucial factor in identifying MDR SBP in the clinical setting. The guidelines highlight the rise in MDR bacteria and the difficulty this presents, requiring a high suspicion for MDR in non-responsive cases, even if they do not provide a unique “MDR diagnosis” indication apart from the conventional SBP diagnosis [7]. 

The sample size was calculated based on 16% of SBP among cirrhotic patients with ascites reported by Grover et al. in 2022. Precision 5%, significance 0.05. With 10% drops out give a sample size 230, Our study included 300 [10].

The sample size was estimated based on the expected prevalence of SBP among cirrhotic patients with ascites reported in previous literature. According to Grover et al., the prevalence of SBP was approximately 16%. Using this proportion (*p* = 0.16) with a desired precision of 5% and a significance level of 0.05,the calculated minimum sample size was 207 patients .To account for an anticipated 10% dropout or incomplete data, the final required sample size was adjusted to 230 patients. Our study included 300 patients, which exceeded the calculated requirement and thereby enhanced the statistical power and reliability of the analyses, particularly for subgroup comparisons such as multidrug-resistant infections and spontaneous fungal peritonitis.

### Statistical analysis

The SPSS program was used to statistically analyze the data (V 13-Windows). P-values less than 0.05 were considered statistically significant in all analyses. Together with percentage and frequency, the data are displayed as value, mean, or range with a 95% confidence interval. To analyze qualitative variables, the chi-square test was used. The mean and standard deviation of quantitative variables (with a normal distribution) were evaluated using the Student t-test.

## Results

The hospital admitted three hundred cirrhotic cases with SP. The age range of the studied cases was 20 to 85 years old, with a mean age of 59.50 years (± 10.08). Males made up the majority of the SP cases that were investigated, and 67.3% of them had moderate ascites. Most cases were classified as being in the Child-Pugh C stage (Table 1). According to the current research, HCV Ab was positive in 84.3% of all cases examined. 86% of the cases that were examined had high MELD scores. The average length of stay in the hospital was 8.87 ± 5.98 days. Just 32.3% of people perished (Table 1).

**Table 1:** Clinical characteristics of patients with spontaneous peritonitis (SP)

**Items**	**patients with SP (n=300)** **No (%)**
Age (years):[Mean± SD]	59.50±10.08
Range	20-85
Sex:	
Males	206 (68.7)
Females	94 (31.3)
Diabetes mellitus	107 (35.7)
Hypertension	54 (18.0)
Hepatocellular carcinoma	144 (48.0)
Abdominal exploration	41 (13.7)
History of paracentesis	50 (16.6)
Hepatic Encephalopathy	134 (44.7)
Portal vein thrombosis	53 (17.7)
Ascites:	
Mild	54 (18.0)
Moderate	202 (67.3)
Marked	44 (14.7)
Child -Pugh class:	
B	45 (15.0)
C	255 (85.0)
Model for end stage liver disease score	
Moderate <20	42 (14)
High ≥20	258 (86)
Duration of hospital stay (days): ):[Mean± SD]	8.87±5.98
Range	1-31
Outcome:	
Died	97 (32.3)
Improved	203 (67.7)

**Fig. 1:**
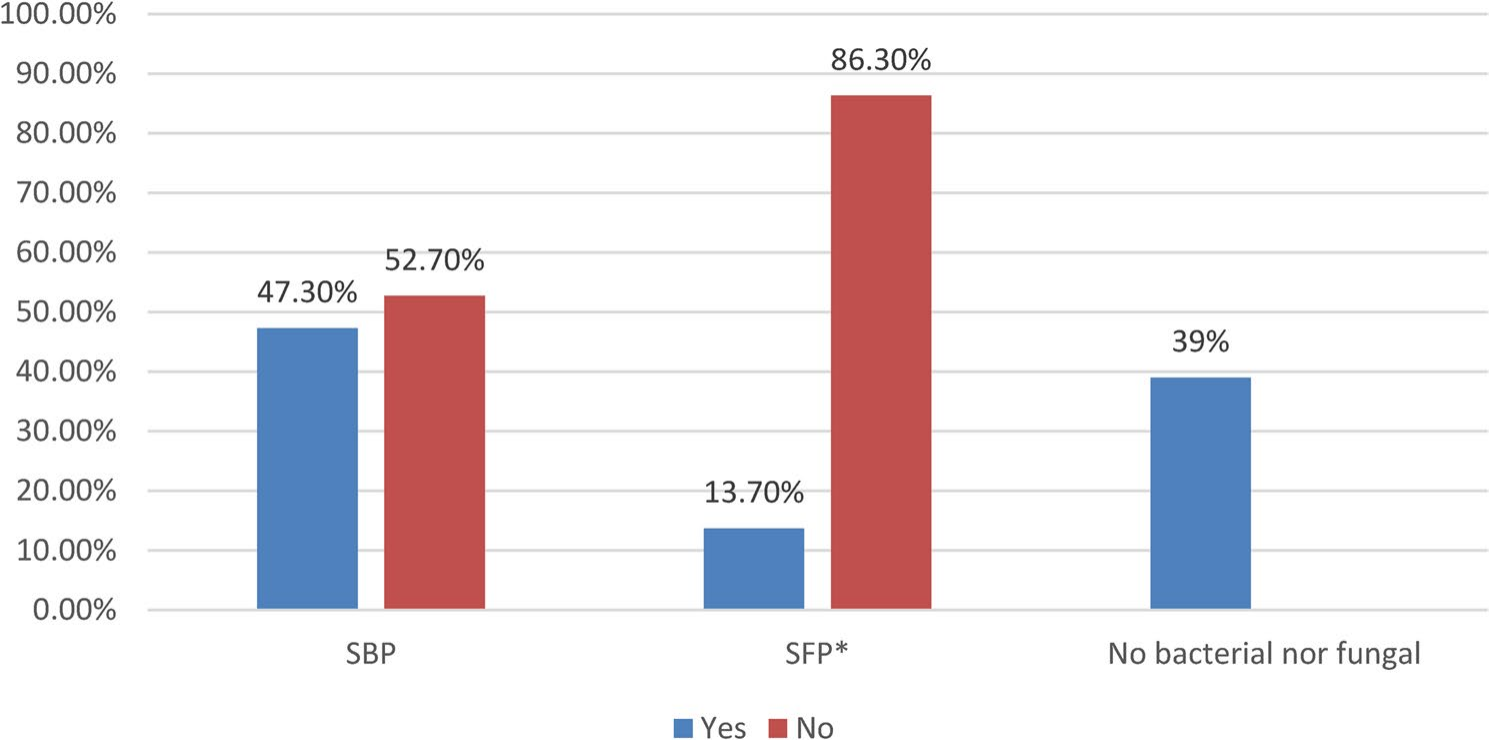
Prevalence of spontaneous bacterial peritonitis (SBP) and spontaneous fungal peritonitis (SFP) among the studied cases

SBP was present in 47.3% of the participants in the research (*n* = 142). 13.7%(*n* = 41) also developed fungal peritonitis on their own. Of the individuals with SP, 10.3% had both SBP and SFP, whereas 39%(*n* = 117) had neither fungal nor bacterial illnesses (Fig. 1).

**Fig. 2:**
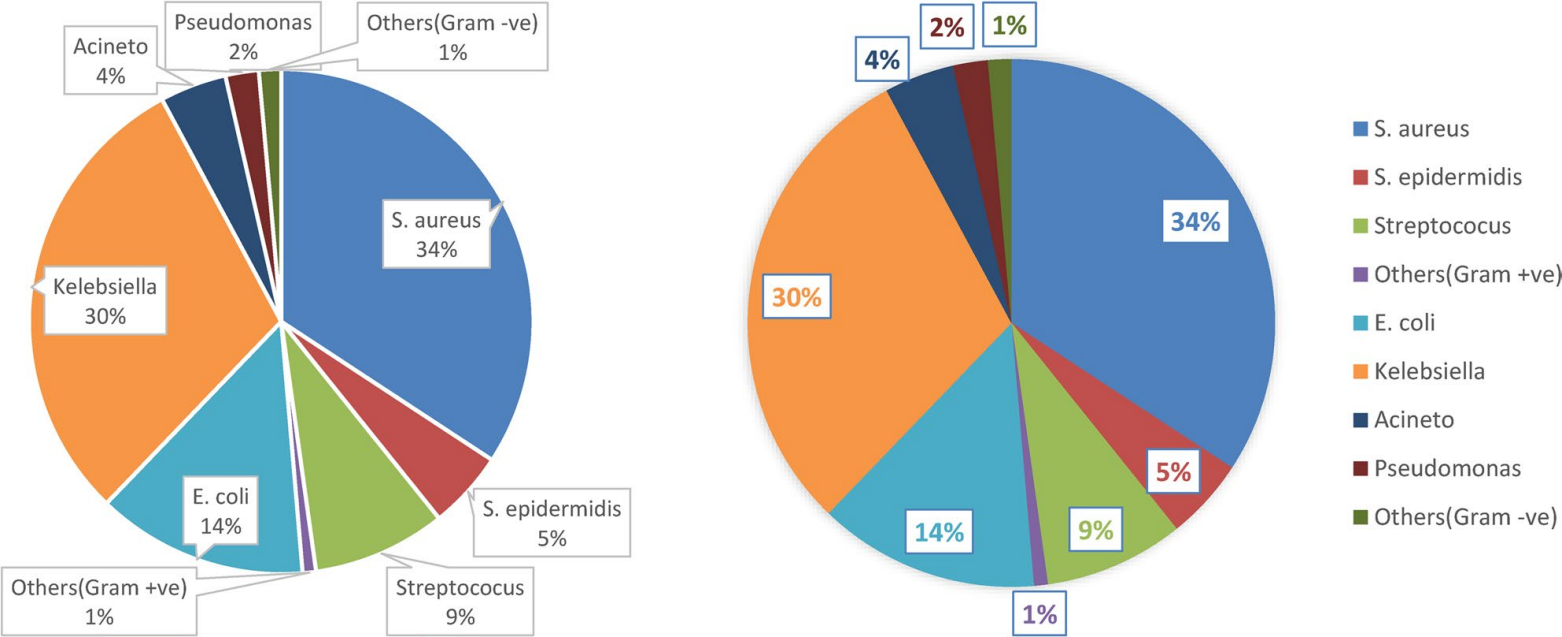
Frequency distributin of patients with SBP as regards types of isolated bacteria

Of SBP cases, 50.7%(*n* = 72) had gram negative (-ve) bacteria isolated from them, whereas 49.3%(*n* = 70) had gram positive (+ ve) bacteria. Streptococcus 8.5% (*n* = 12) was the commenest gram-positive isolate, followed by Staphylococcus aureus 33.8%(*n* = 48). On the other hand, Klebsiella 29.6% (*n* = 42) was the commenest gram-negative isolate, followed by Escherichia coli 14.4%(*n* = 19) (Fig. 2).

**Fig. 3:**
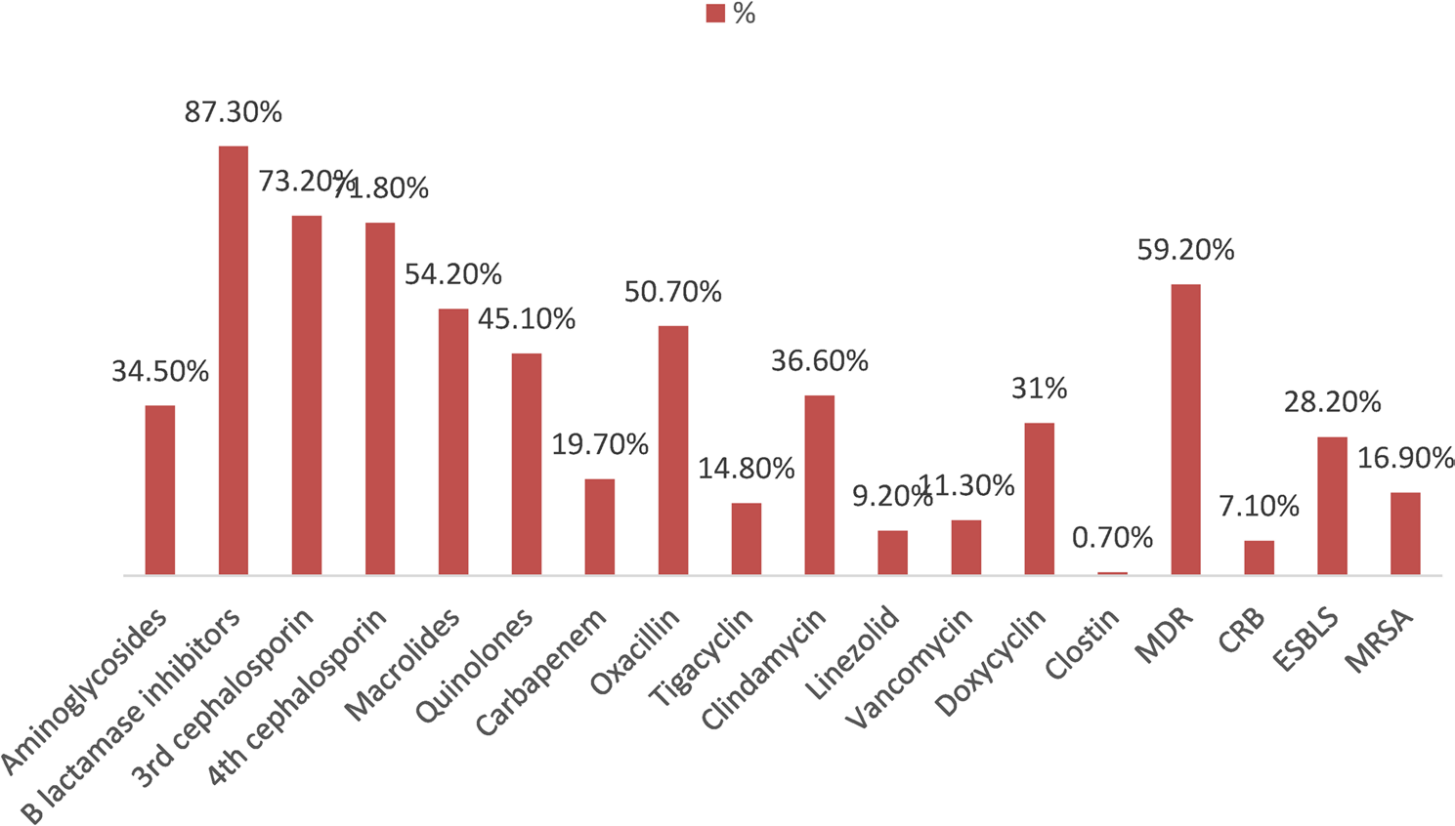
Frequency distribution of patients with SBP as regards the prevalent antibiotic resistance among the isolated bacteria

SBP isolates exhibited the highest resistance to β-lactam antibiotics combined with β-lactamase inhibitors (87.3%, *n* = 124). This was followed by resistance to third-generation cephalosporins (73.2%, *n* = 104) and fourth-generation cephalosporins (71.8%, *n* = 102), macrolides 54.2% (*n* = 77), oxacillin 50.7%(*n* = 72) and others. 59.2% (*n* = 84) of SBP cases acquired MDR (Fig. 3).

According to this table, the gender distribution of cases with and without SFP differed significantly, with the percentage of female cases with SFP being higher than that of cases without SFP (*p* = 0.026) (Table 2). According to this table, cases with SFP had a significantly higher mean value of Na than cases without SFP. There were two (4.9) HBs Ag-positive cases with SFP and fourteen (5.4%) without SFP. Thirty four cases with SFP (82.9) and 219 cases without SFP (84.6%) had HCV (Table 3).

**Table 2:** Clinical characteristics of patients with and without SFP

**Items**	**SP patients**			
	**With SFP ** **(n=41)** **No (%)**	**Without SFP (n=259)** **No (%)**	**χ** **2**	**p-value**
Age (years):				
Mean± SD	60.18±11.08	59.92±9.24	t=0.14	0.890
BMI (kg/m^2^)				
Mean± SD	22.83±2.90	22.40±2.49	t=0.87	0.387
Sex:				
Males	22 (53.7)	184 (71.0)	4.97	**0.026**
Females	19 (46.3)	75 (29.0)		
Diabetes Mellitus	11 (27.5)	31 (30.4)	0.12	0.734
Hypertension	7 (17.1)	47 (18.1)	0.03	0.868
Hepatocellular carcinoma	23 (56.1)	121 (46.7)	1.25	0.264
Abdominal paracentesis	10 (24.4)	40 (15.5)	2.52	0.472
Hepatic encephalopathy	20 (48.8)	114 (44.0)	0.46	0.794
Portal vein thrombosis	9 (22.0)	44 (17.0)	0.60	0.439
Splenectomy	1 (2.4)	6 (2.3)	0.01	0.962
immunosuppressive drugs	0	2 (0.8)0.32	0.32	0.572
Ascites:				
Mild	6 (14.6)	48 (15.8)	0.49	0.782
Moderate	28 (68.3)	174 (67.2)		
Marked	7 (17.1)	37 (13.7)		
Child -Pugh stage:				
B	4 (9.8)	41 (15.8)	1.02	0.312
C	37 (90.2)	218 (84.2)		
MELD score:				
Moderate < 20	3 (7.3)	39 (15.1)	1.76	0.184
High ≥20s	38 (92.7)	220 (84.9)		
Duration of hospital stay (days):[Mean± SD]	7.75±4.22	7.90±4.91	t=0.17	0.864

*SFP* Spontaneous fungal peritonitis, *SD* Standard deviation, *MELD* Model for end stage liver disease

**Table 3:** Laboratory investigations of patients with and without SFP

**Items**	**SP patients**			
	**With SFP** **(n=41)** **Mean± SD**	**Without SFP (n=259)** **Mean± SD**	**t-test**	**p-value**
Hb (g/dl)	10.33±2.10	10.23±2.11	0.29	0.772
Leucocyte 10^9^/L	12.02±6.75	10.67±5.72	1.37	0.173
Neutrophil 10^9^/L	8.61±5.80	7.72±6.01	0.90	0.371
Lymphocyte 10^9^/L	2.72±1.19	2.76±1.50	0.16	0.873
Platelets 10^9^/L	156.59±126.98	151.14±108.39	0.29	0.771
AST (IU/L)	151.83±200.96	118.32±191.06	0.26	0.796
ALT (IU/L)	69.88±82.27	63.88±113.48	1.04	0.30
Albumin (g/dl)	2.14±0.50	2.23±0.51	0.33	0.746
Total bilirubin (mg/dl)	7.40±9.95	5.74±6.67	1.06	0.288
Direct bilirubin (mg/dl)	5.12±7.18	3.99±4.95	1.03	0.171
Prothrombin time (sec)	18.07±4.07	17.29±5.64	1.27	0.207
INR	1.63±0.39	1.59±0.40	0.85	0.396
APTT	48.98±6.25	47.77±7.47	0.67	0.502
S.Alkaline phosphatase (IU/L)	155.68±119.89	144.99±108.36	0.98	0.329
GGT (IU/L)	100.98±140.98	87.92±116.74	0.58	0.563
Urea (mg/dl)	111.02±72.15	100.54±68.40	0.65	0.519
Creatinine (mg/dl)	1.79±1.14	2.00±3.95	0.91	0.366
ESR	34.34±20.81	34.82±24.37	0.33	0.741
CRP	83.82±67.33	69.45±62.60	0.12	0.906
LDH U/L	206.39±150.20	160.64±122.15	1.35	0.178
Sodium mmol/l	127.44±3.69	125.78±5.53	2.15	**0.032**
Potassium mmol/l	4.47±0.86	4.46±0.86	1.86	0.064
HBS Ag positive [No (%)]	2 (4.9)	14 (5.4)	χ2=0.02	0.889
HCV Ab positive [No (%)]	34 (82.9)	219 (84.6)	χ2=0.07	0.780

*SFP* Spontaneous fungal peritonitis, *SD* Standard deviation, *HB* Hemoglobin, *WBCs* White blood cells, *AST* Aspartate aminotransferase, *ALT* Alanine aminotransferase, *INR* International normalized ratio, *APTT* Activated partial thrombosplastine time, *ALP* Alkaline phosphatase, *GGT* Gamma glutamyltranspeptidase, *ESR* Erythrocyte sedimentation rate, *CRP* C-reactive protein, *LDH* Lactate dehydrogenase, *HBs Ag* hepatitis B surface antigen, *HCV Ab* hepatitis C virus antibody

This table demonstrated that the ascitic fluids of cases with SFP had significantly lower mean protein values and significantly higher mean LDH values than those of cases without SFP (Table 4). Of the 41 SFP-positive cases, 20 (48.8%) were dead and 21 (51.2%) were improved, according to this table, which displays the percentage of dead and improved cases (Table 5).

**Table 4:** Laboratory measures of ascitic fluid samples of patients in relation to SFP

Items	SP patients		Mann-Whitney test	*P*-value
	with SFP (*n* = 41) Mean ± SD	without SFP (*n* = 259) Mean ± SD		
Total leucocyte count 10^9^/l	3.25 ± 4.55	3.25 ± 4.56	0.01	0.999
Neutrophil 10^9^/l	2.74 ± 4.33	2.66 ± 4.16	0.11	0.914
Lymphocytes 10^9^/l	1.85 ± 1.62	1.12 ± 0.90	1.01	0.325
Protein g/dl	1.04 ± 0.24	1.16 ± 0.36	2.07	**0.039**
Glucose mg/dl	100.27 ± 31.75	99.80 ± 32.36	0.09	0.931
Lactate dehydrogenase U/L	642.63 ± 686.45	460.71 ± 490.44	2.07	**0.039**

Lymphocyte present in 2 cases with MDR and 7 cases without MDR

**Table 5:** Percent distribution of dead cases among spontaneous fungal peritonitis (SFP)

		Out		Total
		Died	Improved	
SFP	positive	20(48.8)	21(51.2)	41
	negative	77	182	259
Total		97	203	300

Of the 97 cases who passed away, 52 instances (53.6%) had culture-positive results: 20 had SFP (20.6%), 20 had mixed infections (20.6%), and 32 had SBP (33.0%). 45 instances had culture-negative results, meaning that no fungus or bacteria were found .

On multivariate analysis, longer hospital stays and higher haemoglbin (Hb) levels were independent predictors of poor outcomes in SBP with MDR (*p* = 0.026, 0.043, respectively) ( Table 6).

**Table 6:** Binary logistic regression analysis for relevant predictors of the outcome of patients with SBP and MDR

Predictors	B	*p*-value	OR	95% CI	
				Lower limit	Upper limit
Child- Pugh stage C	20.947	0.998	125.827	0	-
Duration of hospital stay (days)	-0.161	**0.026**	0.851	0.739	0.981
HB (gm/dl)	-0.323	**0.043**	0.724	0.529	0.990
Creatinine (mg/dl)	-0.035	0.891	0.965	0.584	1.596
Protein in ascitic fluid (g/dl)	1.839	0.057	6.290	0.944	41.895
Glucose in ascitic fluid (mg/dl)	0.019	0067	1.019	0.999	1.041

*SBP* Spontaneous bacterial peritonitis, *MDR*  Multidrug resistance, *HB*  Hemoglobin

As shown in Table 7, multivariate analysis revealed that femal (*p* = 0.013), low ascitic protein level, and high ascitic LDH (*p* = 0.018, *p* = 0.014) were independent predictors for SFP.

**Table 7:** Binary logistic regression analysis for relevant predictors of the outcome of patients with SFP

Predictors	Univariate				Multivariate			
	B	*P*-value	OR	95% CI	B	*P*-value	OR	95% CI
Gender	0.751	0.028*	2.11	1.08–4.1	0.878	0.013*	2.40	1.20–4.81
Sodium mmol/l	0.076	0.066	0.927	0.855–1.005	-----	-----	----	----
Ascitic Protein g/dl	1.21	0.042*	3.36	1.04–10.8	1.48	0.018*	4.39	1.29–14.9
Ascitic LDH U/L	0.001	0.057*	1.00	99 − 1.00	0.001	0.014*	0.999	0.999-1.00

*SFP*  Spontaneous fungal peritonitis, *LDH*  Lactate dehydrogenase

## Discussion

Three hundred cases with cirrhosis who were hospitalized with SP were included in this cross-sectional investigation. In line with Oliveira et al. [11], whose mean age was 55.2 years and whose sample was 57% male, the mean age of the cases in our study was 59.50 ± 10.08 years, with a male preponderance. In their analysis of 58 SBP cases, Harchand et al. [12] found that the incidence was 51.3% (58/113). The average age of the subjects was 49.72 ± 10.3 years. Men made up 86.7%, while women made up 13.3%, which is consistent with our results.

According to the current research, 84.3% of the cases showed positive HCV Ab and 5.3% had positive HBsAg. This finding is consistent with Setiawan et al. [13], who suggested that the main causes of cirrhosis can vary depending on the region. HBV is predominantly found in Eastern regions while alcoholic liver, HCV and fatty liver are more common in Western countries.

There were 1.09 × 10^9^/L mean lymphocytes, 485.74 U/L mean LDH, 99.86 mg/dL mean glucose, 2.67 × 10^9^/L mean polymorphs, 1.15 g/dL mean protein concentration, and 3.25 × 10^9/L mean total leucocytes in our research, according to the ascitic fluid analysis. These results are consistent with those of Bruns et al. [14], who proposed that cirrhotic patients who have an ascitic total protein content less than 1.5 g/dL are more likely to develop SBP. Additionally, a polymorph count of at least two hundreds and fifty cells/µL in ascitic fluid is indicative of SBP. Runyon [15] discovered that cirrhotic subjects with ascitic protein levels less than 1 g/dL were 10 times more likely to develop SBP than those with higher levels. Ascitic fluid’s opsonic or antibacterial activity showed a substantial correlation with protein contents. As a result, those with low protein levels are more likely to develop SBP. On the other hand, cases who have ascitic fluid with a high protein level such as those who have malignant ascites or congestive heart failure show a relative resistance to SBP. As the most accurate indicator of the first SBP episode, ascitic fluid protein levels have been confirmed by additional research.

About 13.7% of SP people in this research developed SFP, compared to 47.3% who got SBP. According to Gunjača and Francetić [9], the incidence of SBP in hospitalized cirrhotic cases with ascites is estimated to be between 10% and 30%. About 3.5 to 10% of cases with ascites and cirrhosis develop SBP, whereas 5% develop secondary bacterial peritonitis, according to Kuftinec et al. [16]. According to a different research by Fernandez et al. [17], fungal infections happened in nine instances of acute-on-chronic hepatic failure, or 2% of the cohort, and 37% of the cases analyzed developed SBP.

In this investigation, 50.7% of SBP individuals had gram-negative bacteria extracted from them, while 49.3% had gram-positive bacteria. The results run counter to those of Cai et al. [18], who found that the prevalence of gram-positive organisms was higher than that of gram-negative organisms. Ding et al. [19] reported the isolation of 334 pathogenic strains from patients’ ascitic fluids, comprising 18 other microbial strains, 138 Gram-positive bacterial strains, and 178 Gram-negative bacterial strains. E. coli constituted the predominant pathogen at 24.3%, succeeded by Klebsiella pneumoniae (12.0%) and Enterococcus faecium (10.5%).

The predominant Gram-positive bacterium identified in this study was S. aureus, with Streptococcus following as the second most common type. Klebsiella and E. coli were the most prevalent Gram-negative bacteria. This aligns with the findings of Niu et al. [20], which indicate an elevated pathogenic bacteria incidence previously associated with SBP, including *Enterobacter species*, *Klebsiella species*, and *E. coli*.

While Oliveira et al. [11] reported that 62.1% of their cases were classified as Child-Pugh C stage, the majority of cases in this research were classified as such at 85%. According to Ramanathan et al. [21], there is a significant association between the hepatic venous pressure gradient(HVPG) and the grades of CTP classes. Higher ascites risk is associated with an HVPG of more than 18.5 mmHg, whereas the development of SBP has been associated with an HVPG of more than 20 mmHg. Additionally, both nosocomial and community SBP showed a notable incidence of bacteria that were extensively and multidrug-resistant. In a multicenter prospective research on MDR infections, including SBP, Salerno et al. [22] discovered a higher death rate compared to cases with antimicrobial-susceptible infections.

However, in certain studies, antimicrobial resistance had no negative effect on the prognosis of SBP ( [23, 24]). With a P-value of 0.932, our analysis showed that MDR did not significantly differ in outcomes for SBP cases (died-improved). According to a research by Oliveira et al., among cases with SBP, there was no statistically significant difference between those infected with a resistant bacterium and those infected with a nonresistant one in the following outcomes: hospital mortality (57.3% x 58.1%), renal replacement therapy initiation (41.5% x 42.9%), or intensive care unit admission (70.7% x 59.2%) [11]. 

According to Alexopoulou et al., cases with XDR infections had worse outcomes than cases with MDR and those without DR infections individually (log rank *P* = 0.012 and *P* = 0.008, respectively). As a result, the 30-day survival rate for cases with MDR bacteria was comparable to that of cases with non-DR bacteria (log rank *P* = 0.604). The 30-d-mortality was 37.7% overall. Specifically, the 30-day death rate was 69.2% for cases with XDR and 34.2% for the remaining cases, likely due to New XDR strains may have emerged as a result of the widespread use of carbapenems in the majority of cases with nosocomial and health care associated (HCA) infections, which cautions against the overuse of these antimicrobials [25]. 

However, it is necessary to consider the potential for a beta error. However, after controlling for MDR and MELD-Na, the Furey et al. investigation revealed that Streptococcus-associated SBP had the best clinical results and Klebsiella-associated SBP had the worst. Therefore, identifying the causing microbe is essential for both prognostication and therapeutic optimization [26]. 

Cases with SBP who have cirrhosis are more likely to die. High prognosis scores like CTP and MELD-Na at presentation, combined with elevated white blood cell counts and high ascitic fluid lactate levels at presentation, were found to be potential and accurate predictors of mortality in SBP cases in the Kumar et al. research [27]. 

In line with the findings of Oliveira et al. [11], who found no significant correlation between bacterial resistance and the investigated potential risk factors—aside from nosocomial infection—no significant relationship was found between any clinical SBP characteristics with regard to MDR. Previous studies did not show that the severity of cirrhosis or antimicrobial prophylaxis (with norfloxacin) significantly affected the incidence of MDR in SBP ( [28, 29]).

The mortality rate for MDR and SBP cases in this research was 36.9%, but 63.1% of cases showed improvement. 52.2% of the SBP individuals with MDR in the Cai et al. [18] trial passed away. Third-generation cephalosporin resistance, nosocomial infections, and the ineffectiveness of empirical treatment are among the factors that contribute to in-hospital mortality that exceeds 30 days [30].

The commenest kind of drug resistance in SBP was seen with β-lactamase inhibitors, followed by oxacillin, macrolides, third- and fourth-generation cephalosporins, and others. Third-generation cephalosporins are frequently administered as the first empirical treatment in international recommendations, hence the evolution of resistance to these antimicrobials is concerning ( [31, 32]).

According to de Mattos et al., The most prevalent pathogens in SBP are aerobic gram-negative bacteria, including Escherichia coli. Crucially, quinolone resistance affects about 30% of isolated gram-negative bacteria. Studies have also shown that gram-positive bacteria are common pathogens in cirrhosis and infection cases. Up to 27% of SBP cases had methicillin-resistant Staphylococcus aureus, according to certain research. The widespread use of quinolones as a prophylactic measure for SBP and the growing frequency of invasive surgeries to address cirrhosis problems are presently thought to be the causes of this kind of infection [33].

According to Wiest et al., ESBL-producing bacteria have been found to be more common in nosocomial infections in individuals with cirrhosis. Resistance to numerous antimicrobials, such as monobactams and third-generation cephalosporins, is linked to the creation of ESBL. Genes encoding resistance to various antimicrobials, including quinolones, tetracyclines, and antifolates, are frequently carried by ESBL-producing bacteria and are easily dispersed throughout hospitals [34]. 

In MDR and SBP cases, there were no differences in the prevalence of gram-positive and gram-negative bacteria. Although gram-positive infections are becoming more common in cirrhotic people, gram-negative bacteria seem to be mostly responsible for genuine SBP [35, 24].

As hepatic disease worsens, as evidenced by bilirubin levels above 3.2 mg/dl and platelet counts below 98,000/mm, the risks of SBP increase. There is an 11% increase in risk for each MELD scale step [36]. In SBP and MDR cases, the mean platelet count was 129.51 ± 80.07, and the mean total bilirubin level was 6.59 ± 7.90.

According to our research, the average blood creatinine and hemoglobin levels in dead individuals were significantly higher than those in improved MDR cases. About 40% of people with SBP develop renal impairment, which is a significant predictor of mortality in this population. A reduction in effective arterial blood volume is the cause of renal impairment. An effective supplement to antimicrobial treatment is albumin-induced plasma volume expansion, particularly in cases with renal impairment and jaundice [37].

This research identifies female sex, elevated ascitic fluid LDH levels, and decreased ascitic fluid protein levels as the main predictors of poor outcomes in SBP with MDR. These findings are consistent with the research of Wiest et al. [34], which shows that cases who have longer hospital stays and more bacterial translocation are more likely to have poor SBP outcomes.

Additionally, the research finds that female sex, higher ascitic fluid LDH levels, and lower ascitic fluid protein levels are important predictors of SBP.90.2% of the cases under research were assigned to Child-Pugh stage C, which is consistent with Shizuma’s research, which found that 100% of cases were in this stage. Fungal ascites is currently the subject of little investigation. SFP was more common in child C, high MELD, and hepatocellular cancer in our research. Shizuma (2018) found that severe hepatic disease, high MELD or Child-Pugh scores, renal impairment, HCC, long-term antimicrobial use, and delays in diagnosis and treatment are risk factors for SFP in cases with hepatic cirrhosis. Low quantities of ascitic fluid protein and elevated bilirubin levels are additional risks [38]. 

Nine instances of fungal ascites were described by Bucsics et al. [39] who noted that two cases were classed as class C and seven as Child-Pugh class B. Furthermore, the in-hospital death rate was 33%, and bacterial coinfections occurred in 44% of cases (3/9).

Antimicrobial resistance profiles of microorganisms isolated from ascitic fluid in 311 ESLD cases admitted to a German tertiary care during the first SP episodes (2007–2013) were assessed by Friedrich et al. [40]. Of the 138 pathogens found, 89 were nosocomial and 49 were acquired in the community. Ten cases (7.2%) had fungal infections, which were primarily brought on by Candida species, with C. albicans being the commenest isolate (3.6%). The distribution of Candida spp. in nosocomial (9.0%, *n* = 8) and community-acquired (4.1%, *n* = 2) cases did not differ significantly (*P* = 0.287).

Limitations of our research were low numberof cases, a lack of clear symptoms that makes identification difficult, difficulties cultivating fungus, a lack of proven risk factor consensus, limited antifungal susceptibility testing, and differences in diagnostic criteria between investigations. These elements make it challenging to create consistent treatment plans and forecast case outcomes, and they also contribute to an underestimation of the prevalence and impact of SFP.

## Conclusion

SBP and SFP frequently provide serious life-threatening dangers in cirrhotic patients with ascites. To overcome low prevalence and increase statistical power, large multi-center studies should be conducted. Gram-negative bacteria were found to be more common in SBP infections. However, it was also observed that a significant portion of hospital infections and gram-positive microbes were bacteria that were resistant to antimicrobials. Alertness for SFP is increased with low ascitic protein level and high ascitic LDH. Examining efficient antifungal therapy regimens is warranted particularly for mixed infections. 

## Data Availability

Available on request , the datasets used and/or analysed during the current research are available from the corresponding author on reasonable request.

## Abbreviations

CAIDS: Cirrhosis-associated immune dysfunction syndrome
CNNA: Culture-Negative Neutrocytic Ascites
HBV: Hepatitis B virus
HCV: Hepatitis C virus
LDH: Lactate dehydrogenase
MDR: multidrug-resistant
MELD: Model for end stage hepatic disease
PMN: Polymorphonuclear cell
RE: reticuloendothelial
SBP: Spontaneous bacterial peritonitis
SP: spontaneous peritonitis
UTIs: urinary tract infections
WBC: White blood cell
